# An Unusual Presentation of COVID-19 Associated Multisystem Inflammatory Syndrome in Adults (MIS-A) in a Pregnant Woman

**DOI:** 10.1155/2022/4186846

**Published:** 2022-01-07

**Authors:** Mohamed Rishard, Suren Perera, Kushan Jayasinghe, Amila Rubasinghe, Sanjaya Athapaththu, Malindu Edirisinghe, Prabhodana Ranaweera, Tushani Ranawaka, Athula Kaluarachchi, Priyankara Jayawardana, Zacky Haniffa

**Affiliations:** ^1^Department of Obstetrics and Gynecology, Faculty of Medicine, University of Colombo 25, Kynsey Road, Colombo 08, Sri Lanka; ^2^De Soysa Hospital for Women Kynsey Road, Colombo 08, Sri Lanka

## Abstract

Based on available literature, pregnant women are at an increased risk of severe illness from severe acute respiratory syndrome coronavirus 2 (SARS-CoV-2) infection, compared to nonpregnant women. Consequences of coronavirus disease 2019 (COVID-19) in pregnancy have many implications in women's lives other than unfavorable obstetric outcomes. In addition to managing acute respiratory illness and symptoms, caregivers should be equipped to detect and manage the short-term, intermediate, and long-term consequences of SARS-CoV-2 infection as well. Many pregnant women can remain asymptomatic and continue their pregnancy without being diagnosed. Pregnancy outcomes and consequences of SARS-CoV-2 infected yet asymptomatic mothers have not been very well explained. Reports of a new multisystem inflammatory syndrome in children (MIS-C) and multisystem inflammatory syndrome in adults (MIS-A) following COVID-19 have been described. However, MIS-A in pregnancy is an extremely rare presentation that can cause a huge diagnostic dilemma to caregivers. We describe the successful management of a pregnant woman with MIS-A following SARS-CoV-2 infection.

## 1. Introduction

The world's healthcare paradigms have been changed within a short period of time, by the new disease coronavirus disease 2019 (COVID-19) as a result of the novel severe acute respiratory syndrome coronavirus 2 (SARS-CoV-2) viral infection, which has infected millions of people all over the world and caused 2.9 million deaths by April 2021 [1].

Pregnancy and manifestations of COVID-19 are still not well understood. This has led to a huge dilemma among the caregivers. Understanding the spectrum of its manifestations is mainly based on case reports, and new information is added to existing literature on this area.

At the beginning, it was thought that pregnant women were not at higher risk than nonpregnant women [2]. However, in a multinational cohort study, COVID-19 in pregnancy was associated with significant increase in severe maternal morbidity and mortality. It has been shown that pregnant women with COVID-19 are at 22 times increased risk of maternal death [3].

Although many recover from acute infection, some patients experience the consequences requiring further follow-up and specialized care. These post-COVID-19 conditions may present with many overlapping symptoms, and correct diagnosis may become a huge challenge. Moreover, they may present with or without any evidence of overt SARS-CoV-2 infection. Multisystem inflammatory syndrome in children (MIS-C) and multisystem inflammatory syndrome in adults (MIS-A) following COVID-19 have been described [4]. MIS-A is defined by extrapulmonary organ dysfunction and absence of respiratory illness [4]. Although outcomes of MIS-A in pregnant women following pregnancy are not well known, this condition leads to a critical condition requiring multidisciplinary team involvement. We present an unusual presentation of MIS-A following SARS-CoV-2 infection in pregnancy.

## 2. Case Presentation

A 28-year-old pregnant mother in her second pregnancy was transferred at 30 + 6 weeks of gestational age with a history of acute onset high grade fever with chills and difficulty in breathing for a period of two days. Her antenatal period had been uneventful up until then. She had delivered by cesarean section due to pregnancy induced hypertension in the first pregnancy.

On examination, she was febrile; her pulse rate was 140 beats per minute and had a respiratory rate of 40 cycles per minute. Lung bases were clear.

Rapid antigen test for SARS-CoV-2 showed a negative result, and three consecutive samples for SARS-CoV-2 reverse transcriptase polymerase chain reaction (SARS-CoV-2 RT-PCR) resulted inconclusive. She was managed in the high dependency unit under multidisciplinary care. Since she was breathless, high flow oxygen was given along with other supportive measures. From fourth to eighth day of illness, she became increasingly tachycardic and tachypneic with high fever, left-sided cervical lymphadenopathy, and rising inflammatory markers. White blood cell count was 13 × 10^9^/L; platelet count was 120 × 10^9^/L. Blood picture revealed an absolute lymphocytosis. Her blood and urine cultures showed no significant growth. 2D echocardiogram showed a grade two diastolic dysfunction with an ejection fraction of 50% with a mild pericardial effusion and elevated troponin I (0.401 ng/L). Her chest X-ray showed a mild pleural effusion. She was not in a position to mobilize for computed tomography scan of the chest. She was commenced on intravenous Ceftriaxone and Oseltamivir despite sterile blood culture reports. SARS CoV-2 specific total antibody including IgG became positive indicating a previous exposure. Her initial arterial blood gas revealed a compensated respiratory alkalosis. She was able to maintain a saturation of 95% on room air. Her liver and renal functions remained normal throughout.

Despite repeated blood, urine, and sputum cultures being sterile, her procalcitonin levels were elevated 2.03 ng/mL (normal < 0.5), CRP was 321, and ESR was 120 mm in the 1st hour. Cardiologist reconfirmed cardiac dysfunction with an ejection fraction of 50%, with mild pericardial effusion, elevated troponin I (0.045 ng/mL), and elevated BNP levels (335 pg/mL). With the evidence of myocardial injury, digoxine, bisoprolol, and asprin were added. Her lower limb compression Doppler was normal, and 2D echo was negative for right heart strain; hence, computed tomography pulmonary angiogram (CTPA) was not carried out considering the risks and clinical status. She was investigated for dengue, influenza, HIV, typhoid, legionella, mycoplasma, leptospirosis, connective tissue disorders, and excluded.

However, she was commenced on therapeutic dose of low molecular weight heparin. By day 12 of illness, she improved remarkably. She was mobilized and discharged. One week later, she presented with gradual onset left-sided lower motor type facial nerve palsy which subsided with a course of steroids and physiotherapy.

During the course of her management, it became evident that this was a novel clinical presentation of MIS-A. On detail inquiry, she recalled a possible COVID-19 exposure 4 weeks ago, when she came in contact with a few people who had fever; however, their COVID-19 status was unconfirmed.

At 37 weeks of gestation, she developed severe pregnancy induced hypertension and delivered a baby boy weighing 2.46 kg by a cesarean section.

## 3. Discussion

It is not yet clear how and why adults develop MIS-A following SARS-CoV-2 infection. However, it is believed to be due to SARS-CoV-2-induced hyperinflammatory reaction [5]. Extrapulmonary dysfunction in COVID-19 is believed to occur as a result of damage to the endothelium and thromboinflammation and disruption to the regulatory mechanisms of immune responses and renin angiotensin-aldosterone system [6] Table 1.

The temporal relationship between SARS-CoV-2 infection and MIS-A is not clear. However, a case series states that 2-5 weeks following typical COVID-19 symptoms and symptoms of MIS-A could occur [7]. Our patient could recall a potential exposure 4 weeks prior; this was tallying with her serological markers too. A repeatedly inconclusive result on multiple SARS-CoV-2 RT-PCR samples may be obtained due to a virological cause such as when either one of the two targets but not both are above the threshold for positivity or when there is some reactivity in the assay which may not suffice to yield a positive result or nonspecific binding of PCR primer during the late phases of PCR cycles [8]. Thus, a clinical correlation of the symptoms, history of exposure, and serological evidence of antibodies of this unvaccinated lady is likely to depict a past infection of COVID-19.

Features to diagnose MIS-A include “a severe illness requiring hospitalization in a person aged ≥21 years; a positive test result for current or previous SARS-CoV-2 infection (nucleic acid, antigen, or antibody) during admission or in the previous 12 weeks, severe dysfunction of one or more extra pulmonary organ systems (e.g., hypotension or shock, cardiac dysfunction, arterial or venous thrombosis or thromboembolism, or acute liver injury); laboratory evidence of severe inflammation (e.g., elevated CRP, ferritin, D-dimer, or interleukin-6); and absence of severe respiratory illness (to exclude patients in which inflammation and organ dysfunction might be attributable simply to tissue hypoxia)” [7]. Our patient presented with acute onset shortness of breath with evidence of myocarditis and laboratory evidence of severe inflammation and had met all diagnostic criteria of MIS-A.

At the time of initial presentation, her clinical presentation was complex. Initially, her symptoms mimicked the clinical picture of acute pneumonia or pulmonary embolism. Then, with the evidence of cardiac injury and elevated inflammatory markers, nonsignificant changes in chest X-ray, the working diagnosis was changed in the line of MIS-A. This is not an unusual situation as they present with a heterogeneous clinical picture. Similarly, many factors can lead to a delayed diagnosis in adults. These include less severe cardiac involvement, negative diagnostic testing at the time of the presentation, limited diagnostic role for antibody testing, diagnostic difficulty with preexisting cardiac disease, COVID-19-related acute myocardial injury having a higher incidence, and multiple causes.

According to data available, many had multiple negative SARS-CoV-2 RT-PCR during cardiac presentations (mean 4.6), and some had a recent history of COVID-19 and recovery afterwards [9]. In one case series, out of 11 cases of MIS-A patients, 10 patients showed positive SARS-CoV-2 RT-PCR test results; seven of them had positive antibodies during the same time [7].

Although serological testing is an option in patients with negative SARS-CoV-2 RT-PCR tests, kinetics of serological markers is not well known. However, there is evidence to show that supplementing with serological tests when there is a negative or unequivocal test could help in the management of patients as well managing contacts [10]. Tests to quantify serum specific antibodies against SARS-CoV-2 are not freely available in all settings. This could be a big challenge in settings where prevalence of COVID-19 is high. This could mean that caregivers should empirically commence treatment in such cases without depending on the antibody status if there is a strong suspicion of multisystem involvement even without evidence of acute SARS-CoV-2 infection. In our patient, high values of cardiac markers and 2D echocardiogram findings were adequately favoring the diagnosis of myocardial injury.

Another useful distinguishing feature is thrombocytopenia which occur as a result of mediators being secreted in the process of eradication of the virus (mainly to stimulate CD8+ cells to kill viral infected cells), which would inadvertently suppress bone marrow function and activate platelets [11]. However, our patient's complete blood count did not show a significant drop in the platelet count.

Many hospitalized COVID-19 patients suffer from myocardial injury. It is believed that the SARS-CoV-2 virus can directly affect the heart. Injury which indirectly occur due to an inflammatory cascade during SARS-CoV-2 infection can also cause cardiac damage. The extent depends on the viral load, the capacity of the host to mount an immune response, and presence of other comorbidities [12]. However, myocardial injury in pregnancy following COVID-19 has been not being well reported. Various physiological changes that occur in pregnancy may make the women more vulnerable to cardiac injury [13]. One report suggests that myocardial injury and left ventricular dysfunction in pregnant women pose a significant risk of maternal death up to 13.3% due to malignant arrhythmias [14]. When positive troponin concentrations are found, it indicates severe illness and poor outcomes in patients with COVID-19. These patients are five times more likely to need ventilation and suffer arrhythmias [15]. Hence, caregivers treating pregnant women with COVID-19 must screen for possible myocardial injury and follow them up in order to avoid serious complications. This would require a huge workforce in low resource settings and may require careful evaluation and selection criterion before requesting cardiac assessment for COVID-19 affected pregnant women. Troponin elevation and high BNP levels in COVID-19 are associated with adverse outcomes and mortality. Values of these two markers should play a key role when managing pregnant women with COVID-19.

Given the associated risks of venous thromboembolism in pregnancy, we commenced therapeutic dose of low molecular heparin. Chau et al. in their case series had commenced therapeutic dose of low molecular heparin in 7 cases [16]. Morris et al. reported that in their case series almost all adults had cardiac dysfunction as a feature and most commonly treated with intravenous immunoglobulin, corticosteroids, and inotropes or vasopressors [7].

Multisystem inflammatory syndrome in adults and in pregnant women has improved with immunoglobulin therapy and corticosteroids [17]. In one case series, rapid and profound improvement in cardiac function occurred soon after initiation of supportive, antimicrobial, or immunomodulatory therapy [10]. In our case, we commenced intravenous broad-spectrum antibiotic and antiviral treatment despite sterile cultures. This was empirical rather than evidence based. However, we believe that we could have commenced steroids as well. Our suspicion about ongoing septic focus prevented us from starting steroids in our patient.

There are reports of facial nerve palsy around the time of SARS-CoV-2 infection and even one month after diagnosis of SARS-CoV-2 infection. This is believed to occur due to an autoimmune pathogenesis [18].

Our patient presented with a complex clinical picture of sepsis, cardiac injury, and Bell's palsy following possible asymptomatic COVID-19, and she met all the diagnostic criteria for MIS-A. All this multifaceted pathophysiology and multiorgan involvement require multidisciplinary team involvement.

There are reports that shows, women who tend to endure COVID-19 symptoms are specially those who are living with social deprivation and those with preexisting comorbidities [19]. This indicates that pregnant women should be educated regarding symptoms of COVID-19, and adequate supportive measures should be provided when they are found to be positive for SARS-CoV-2. This should include psychological support and family support.

Although our patient's clinical condition required an intensive care unit, the team was left with the choice of managing her in the high dependency unit setting due to unavailability of an ICU bed. This situation may prevail or even worsen in the future. She developed severe pregnancy induced hypertension and had to undergo caesarean section. This can be very well attributed to already known pregnancy outcomes of COVID-19.

Clinical vigilance, prompt diagnosis using available resources, and multidisciplinary team involvement are key factors in managing COVID-19-related complications in low resource settings. Caregivers in the primary care services including general practitioners should expect more cases with similar clinical pictures in future. A uniform centralized reporting system is needed to collect data regarding various COVID-19-related conditions to enlighten the caregivers with correct information.

## 4. Conclusion

Clinicians should have a high level of clinical suspicion of MIS-A in any patient presenting with unusual multisystem involvement. Features of myocarditis, fever, and elevated inflammatory markers should prompt the caregivers to correlate with COVID-19 specific serology in the absence of positive SARS-CoV-2 RT-PCR results or obvious history of infection.

High inflammatory markers and fever should not delay the commencement of steroids or immunotherapy. Although antigen, RT-PCR, or serology is mandatory for diagnosis of MIS-A, in the context of a resource limited setup, it may be prudent to start empirical treatment for cases with strong clinical suspicion, preventing undue delays in diagnosis and prompt treatment with steroids and immunoglobulins.

BNP and troponin levels will yield valuable information in pregnant women with cardiac involvement.

Clinicians also should be aware to take in to consideration the physiological changes in pregnancy when interpreting COVID-19-related investigations and clinical parameters that are better described for the nonpregnant population.

Distinguishing the overlapping symptoms of this new disease entity from other medical conditions affecting gravid women can be very challenging and may lead to a diagnostic dilemma. Hence, a good knowledge of various presentations of COVID-19, careful evaluation of the clinical picture, understanding the physiological changes of pregnancy, and their influences on various investigations are vital in managing COVID-19-related complications.

## Figures and Tables

**Table 1:** Summary of investigations.

	Day 0	Day 3	Day 7	Reference ranges
Hemoglobin (g/dL)	11.4	9.3	9.2	11.0–16.0
Platelets (10^3^/*μ*L)	120	187	377	150-450
White blood cells (10^3^/*μ*L)	13.6	18	19	4-10
C reactive protein (mg/dL)	321	291	43.4	<6
Erythrocyte sedimentation rate (ESR) 1st Hr in mm	98	120		
Procalcitonin (ng/mL)		2.03		<0.5
Serum ferritin (ng/mL)		171.5		12-190
High sensitive D-dimers (ng/mL)		1249		<250
High sensitive troponin I (ng/mL)	0.401	0.045		<0.015
Brain natriuretic peptide (BNP) (pg/mL)		335		<28
Elevated liver enzymes	No	No		
Serum creatinine (micromol/L)	56	62	49	49-95
ECG	Sinus tachycardia	Sinus tachycardia	Sinus rhythm	
2D echo	Grade two diastolic dysfunction with an ejection fraction of 50% with a mild pericardial effusion, possible myocarditis	EF 50%, thin rim of effusion	Good biventricular function, no trans thoracic echo evidence of pulmonary embolism Ejection fraction 55-60%	
Chest X-ray	Mild left pleural effusion	Improved		

## Data Availability

All anonymized data are found in the manuscript.
